# Anti-Apoptotic Effect of Apelin in Human Placenta: Studies on BeWo Cells and Villous Explants from Third-Trimester Human Pregnancy

**DOI:** 10.3390/ijms22052760

**Published:** 2021-03-09

**Authors:** Ewa Mlyczyńska, Małgorzata Myszka, Patrycja Kurowska, Monika Dawid, Tomasz Milewicz, Marta Bałajewicz-Nowak, Paweł Kowalczyk, Agnieszka Rak

**Affiliations:** 1 Laboratory of Physiology and Toxicology of Reproduction, Institute of Zoology and Biomedical Research, Jagiellonian University in Krakow, 30-387 Krakow, Poland; ewa.mlyczynska@doctoral.uj.edu.pl (E.M.); m.myszka@student.uj.edu.pl (M.M.); patrycja.kurowska@doctoral.uj.edu.pl (P.K.); monika.dawid@student.uj.edu.pl (M.D.); 2 Department of Gynecological Endocrinology, Jagiellonian University Medical College, 31-501 Krakow, Poland; milewicz@interia.eu; 3 Department of Gynecology and Oncology, Jagiellonian University Medical College, 30-688 Krakow, Poland; marta.balajewicz@gmail.com; 4 Department of Animal Nutrition, The Kielanowski Institute of Animal Physiology and Nutrition, Polish Academy of Science, 05-110 Jabłonna, Poland; pawelkowalczyk1410@gmail.com

**Keywords:** apelin, apoptosis, oxidative stress, placenta, villous explants

## Abstract

Previously, we demonstrated the expression of apelin and G-protein-coupled receptor APJ in human placenta cell lines as well as its direct action on placenta cell proliferation and endocrinology. The objective of this study was to examine the effect of apelin on placenta apoptosis in BeWo cells and villous explants from the human third trimester of pregnancy. The BeWo cells and villous explants were incubated with apelin (2 and 20 ng/mL) alone or with staurosporine for 24 to 72 h. First, we analysed the dose- and time-dependent effect of apelin on the expression of apoptotic factors on the mRNA level by real-time PCR and on the protein level using Western blot. Next, we checked caspase 3 and 7 activity by Caspase-Glo 3/7, DNA fragmentation by the Cell Death Detection ELISA kit and oxygen consumption by the MitoXpress-Xtra Oxygen Consumption assay. We found that apelin increased the expression of pro-survival and decreased proapoptotic factors on mRNA and protein levels in both BeWo cells and villous explants. Additionally, apelin inhibited caspase 3 and 7 activity and DNA fragmentation in staurosporine-induced apoptosis as also attenuated oxidative stress by increasing extracellular oxygen consumption. The antiapoptotic effect of apelin in BeWo cells was mediated by the APJ receptor and mitogen-activated protein kinase (ERK1/2/MAP3/1) and protein kinase B (AKT). The obtained results showed the antiapoptotic effect of apelin on trophoblast cells, suggesting its participation in the development of the placenta.

## 1. Introduction

The placenta is a transient organ whose main function is to ensure optimal conditions for foetal development; it provides the nutrients and oxygen from maternal circulations to the foetus and is the place of the production of numerous hormones necessary for the maintenance of the pregnancy. The development of such an organ must occur within just a few weeks. Thus, during this process, the initial rapid proliferation and invasion of trophoblast cells are followed by their differentiation into individual layers: cytotrophoblast and syncytiotrophoblast. At the same time, the apoptosis of non-functional trophoblast cells must occur [1]. Many reports have suggested that apoptosis in the placenta is a natural physiological phenomenon. It occurs during syncytial fusion, then throughout the remodelling of the entire placenta, when blood vessels are formed to ensure proper blood flow through the tissue, and finally, it is a part of the natural aging of the tissue [2].

Apoptosis, the main type of programmed cell death, is a complex, dynamic and tightly controlled process. It can be activated by two main pathways: external (death receptor pathway) and internal (mitochondrial pathway). In the external pathway, apoptosis-inducing factors act by binding to death receptors, such as tumour necrosis factor receptor (TNFR), or nucleotide-binding oligomerisation domain containing protein 1 (NOD1) and activate receptor domains such as caspase and RIP adapter with death domain (CRADD) or death effector domain containing (DEDD) for further transduction of the death signal [3]. In the intrinsic pathway, B-cell lymphoma 2 family proteins (BCL2) play a key role. They are related to the integrity of mitochondrial membranes, including antiapoptotic BCL2, B-cell lymphoma 3 protein (BCL3), induced myeloid leukaemia cell differentiation protein (MCL-1) and proapoptotic Bcl-2-like protein 4 (BAX), Bcl-2 homologous antagonist/killer (BAK), BH3-interacting domain death agonist (BID) or Bcl2-related ovarian killer protein (BOK) [4]. Nevertheless, both pathways lead to the activation of the caspase cascade, first by initiating caspases 2, 8, 9 and then executive caspases such as 3, 7 or 14, leading to the destruction of cellular components, DNA fragmentation and, finally, to cell death [5]. In addition, several other apoptotic regulators are involved in both the activation pathway and crosstalk between them, such as apoptotic protease-activating factor 1 (APAF1), primarily responsible for creating apoptosome, Diablo IAP-binding mitochondrial protein (DIABLO), or factors inhibiting apoptosis, such as X-linked inhibitor of apoptosis (XIAP) and baculoviral IAP repeat-containing protein 6 (BIRC6) [3].

Although apoptosis is an intensively studied process in the placenta, and also in the context of pregnancy pathologies, there are still some contradictions. Most of the reports indicate that the rate of trophoblast apoptosis increases during pregnancy and is highest in the third trimester of pregnancy, which may be due to natural tissue aging [6,7,8]. On the other hand, other studies found that apoptosis is most pronounced at the beginning of pregnancy in the first trimester and is related to placenta morphogenesis [9,10]. Nevertheless, it is generally considered that apoptosis in the placenta of pregnancies complicated by pathologies, including preeclampsia, intrauterine growth restriction (IUGR) and miscarriage, occurs more frequently than in trophoblast cells from normal pregnancies. In placentas affected by these pathologies, the downregulation of BCL2 expression has been observed, along with higher expression levels of protein p53 and caspase 3 [11,12,13]. In general, apoptosis is mainly observed in the syncytial layer of the placenta, while it is rare in cytotrophoblast cells, possibly because the syncytiotrophoblast consists of the greater part of the placenta and its external part of the trophoblast is more sensitive to death signals [14,15]. Interestingly, Erel et al. [11] observed that increased cell death in placentas from pregnancies complicated with IUGR relative to control subjects occurs mainly in cytotrophoblast cells. The authors suggest that the increased number of apoptotic cells plays an important compensatory role in the transmission of nutrients to the foetus and in gas exchange [11].

Among the numerous factors that regulate the process of apoptosis, adipokines—adipose tissue hormones—are also considered significant regulators of trophoblast survival. For instance, leptin reduces cell death in placental villous explants in a hypothermia model by regulating the p53 pathway [16] and under other pathological conditions including exposure to low pH or serum deprivation [17,18], indicating an important role in trophoblast survival. In contrast, adiponectin has an opposite effect and induces the apoptosis of placental cells [19,20].

Apelin, one of the adipokines, is an endogenous ligand for the G-protein-coupled receptor APJ. It is derived from prepropeptide, which is proteolytic-cleavaged into smaller fragments. The biologically active forms of apelin are 17-, 36-aminoacid fragments and pyroglutamylated apelin-13 (pyr-apelin-13) [21]. Apelin is a pleiotropic peptide involved mainly in the regulation of energy metabolism, fluid homeostasis and angiogenesis [21,22,23]. The presence and function of the apelin/APJ system have also been described for the human placenta [24,25,26]. So far, the role of apelin in facilitating the transport of amino acids and glucose to the foetus, the regulation of hormone secretion and foetal angiogenesis has been demonstrated [27,28,29,30]. In our previous study, we demonstrated the expression of apelin and its receptor APJ in the human placenta cell line JEG-3 and BeWo, as well as its direct action on placenta cell cycle progression and proliferation [26]. Until now, numerous studies have indicated that apelin has an antiapoptotic effect in different cell types. It protects rat adrenal medulla, the human brain, osteoblasts or vascular smooth muscles from apoptosis [31,32,33]. In brain and vascular smooth muscle cells, its antiapoptotic effects are mediated mainly by the APJ receptor and activation on mitogen-activated protein kinase (ERK1/2/MAP3/1) and protein kinase B (AKT) [32,33]. Apelin also has the potential to attenuate oxidative stress, which is linked with apoptosis, as shown in brain cells, adipocytes and bone marrow-derived mesenchymal stem cells [34,35,36]. However, the effect of apelin on apoptosis in human placental cells has not yet been investigated.

In this context, we investigated *(i)* dose- and time-dependent in vitro effects of apelin on the mRNA and protein expression of several apoptotic proteins, caspase 3/7 enzyme activity and levels of histone-associated DNA fragments in BeWo cells; *(ii)* the effect of apelin on oxidative stress in BeWo cells; *(iii)* the involvement of the APJ receptor and kinases MAP3/1 and AKT on apelin-mediated apoptosis in BeWo cells; and *(iv)* dose- and time-dependent in vitro effects of apelin on BCL2/BAX and caspase 3 protein expression as well as levels of histone-associated DNA fragments in villous explants from the human placenta.

## 2. Results

### 2.1. Effect of Apelin on mRNA and Protein Expression of Apoptotic Factors in BeWo Cells

We observed that apelin in placenta BeWo cells modulates, in a dose-dependent manner, the gene expression of multiple factors which are involved in the regulation of programmed cell death. We noted that the mRNA expression of pro-survival *MCL1* and *BIRC6* was significantly increased after apelin at a dose of 2 ng/mL, while the expression of *BCL2* and *BCL3* was decreased (* *p* < 0.05, ** *p* < 0.01, *** *p* < 0.001, Table 1). Moreover, apelin at a dose of 20 ng/mL increased the mRNA level of *XIAP* and *BCL3* expression. The mRNA levels of proapoptotic factors, including *BAK1, BAX, BOK, NOD1, CRADD,* tumour necrosis factor receptor superfamily member 25 *(TNFRSF25)*, the precursor of *caspases 14, 3, 2* and *8*, were significantly lower after the stimulation of apelin at 2 ng/mL. We observed that apelin at a dose of 20 ng/mL also has an inhibitory effect on the mRNA expression of *APAF1, BAK1, BAX, NOD1, DIABLO, TNFRSF25* and the precursor of *caspases 14*, *2*, *3*, *8* and *9* (* *p* < 0.05, ** *p* < 0.01, *** *p* < 0.001, Table 1).

**Figure 1:**
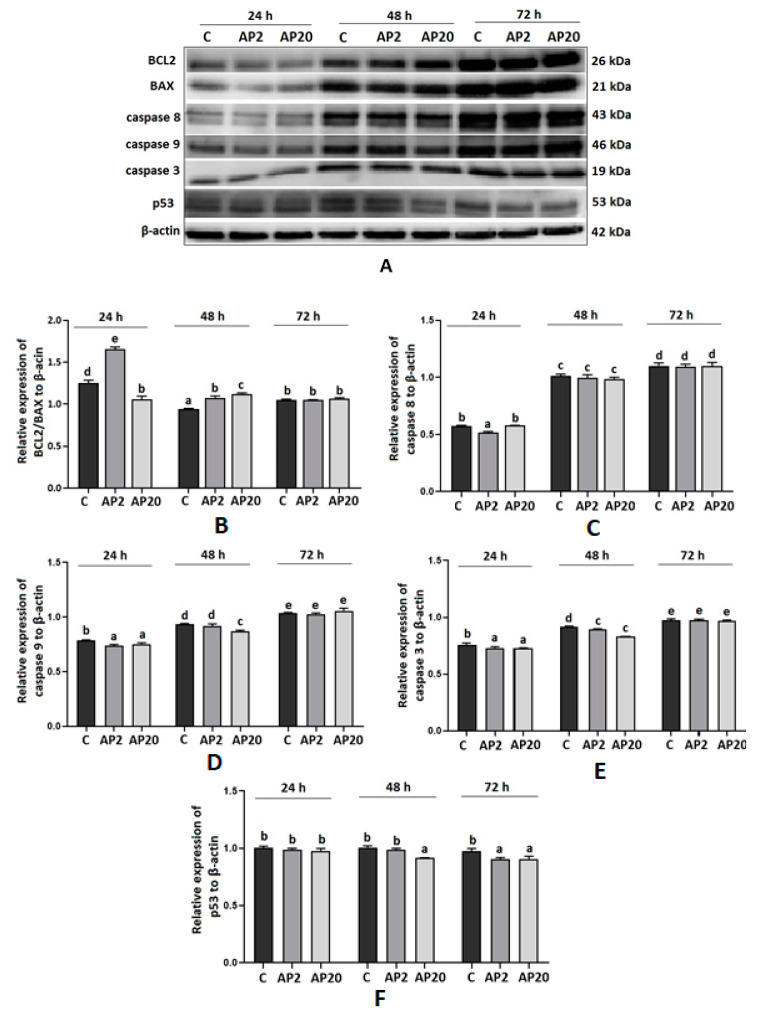
Effect of apelin on protein expression of apoptotic factors in BeWo cells. The cells were incubated with apelin at doses 2 of (AP2) and 20 (AP20) ng/mL for 24, 48 and 72 h, and subsequently, Western blot analysis was performed to examine the expression of BCL2 (B-cell like lymphoma 2), BAX (Bcl-2-like protein 4), caspase 3, 8 and 9 and p53. Results are shown as stripes on gel image (**A**) and densitometry analysis relative to β-actin (**B**–**F**). Experiments were independently performed and repeated three times (*n* = 3). The data are arranged as means ± SEM. Different letters indicate significant differences (*p* < 0.05) among groups; Control (**C**).

Based on the results obtained using real-time PCR, we analysed protein expression by Western blot in apelin-treated BeWo cells. Figure 1 shows that apelin significantly increased the ratio of pro-survival BCL2 to proapoptotic BAX protein expression after 24 h of incubation with 2 ng/mL and after 48 h in both 2 and 20 ng/mL (*p* < 0.05). Moreover, we noticed apelin influence on the protein expression of proapoptotic caspase 8, 9 and 3 by decreasing their expression after 24 and 48 h of incubation. We demonstrated that apelin has no effect on the protein expression of BCL2, BAX and caspases after 72 h of incubation. Interestingly, the protein expression of p53 was significantly decreased after apelin treatment for 48 h at 20 ng/mL and 72 h at both 2 and 20 ng/mL (*p* < 0.05, Figure 1).

### 2.2. Effect of Apelin on Caspase 3/7 Activity and DNA Fragmentation in BeWo Cells

**Figure 2:**
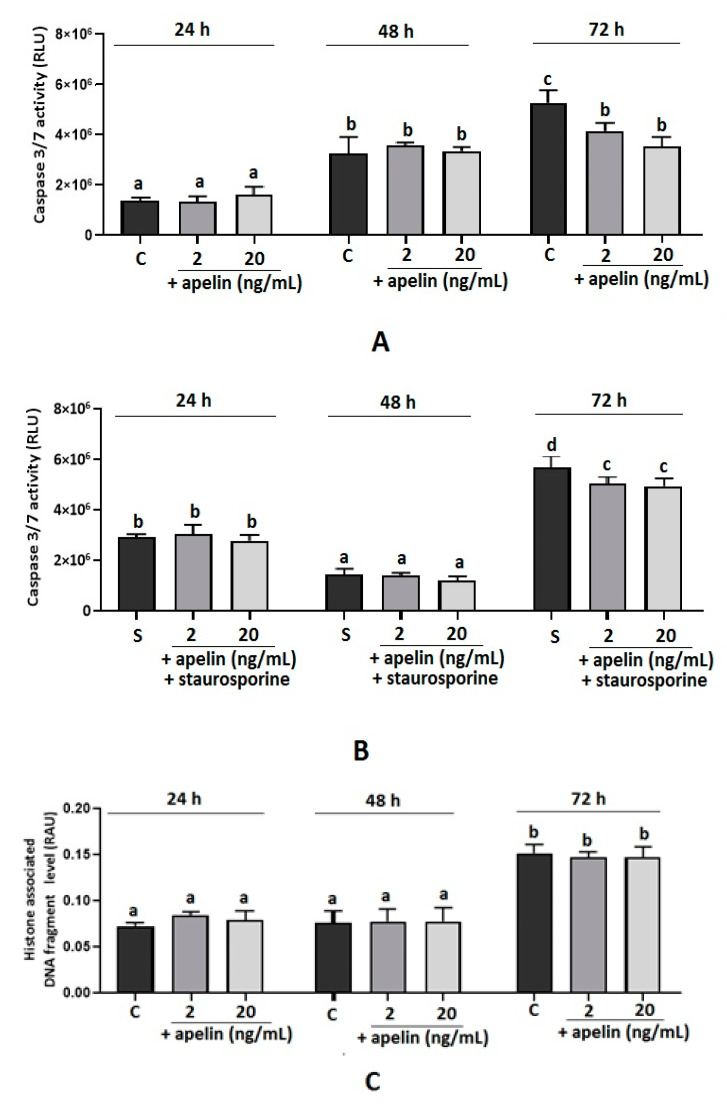

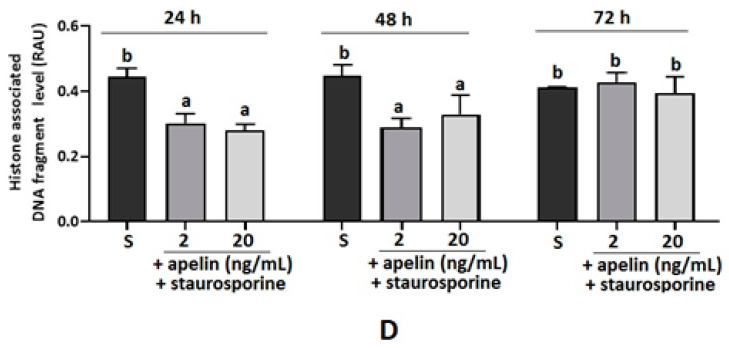
Effect of apelin on caspase 3 and 7 activity and histone-associated DNA fragment level in BeWo cells. The cells were incubated with 2 and 20 ng/mL of apelin alone or in combination with 0.1 µL/mL staurosporine for 24, 48 and 72 h, after which caspase 3 and 7 activity (**A**,**B**) was analysed using the Caspase-Glo 3/7 assay or the level of histone-associated DNA fragments (**C**,**D**) by the Cell Death Detection ELISA kit. Experiments were independently performed and repeated three times (*n* = 3). The data are arranged as means ± SEM. Different letters indicate significant differences (*p* < 0.05) among groups; Control (**C**), Staurosporine (S), Relative Luminescence Unit (RLU), Relative Absorbance Unit (RAU).

A statistically significant inhibition of caspase 3 and 7 activity was observed after 72 h of incubation with apelin at 2 and 20 ng/mL alone and stimulated with staurosporine (*p* < 0.05, Figure 2A,B); no effect was observed after 24 and 48 h of cell incubation. Moreover, we noted that apelin significantly decreased the level of histone-associated DNA fragments in cells incubated with staurosporine after 24 and 48 h of cell incubation (*p* < 0.05, Figure 2C,D). No effect was observed in apelin-treated cells after 24, 48 and 72 h of incubation or apelin with staurosporine after 72 h of incubation.

### 2.3. Effect of Apelin on Oxidative Stress in BeWo Cells

**Figure 3:**
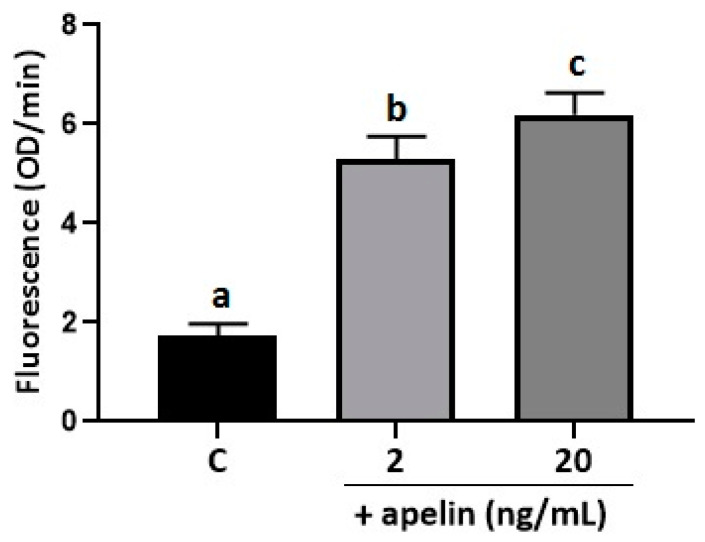
Effect of apelin on oxidative stress in BeWo cells. The cells were treated with apelin at 2 and 20 ng/mL for 24 h, and the oxygen consumption assay was performed. Experiments were independently performed and repeated three times (*n* = 3). The data are arranged as means ± SEM. Different letters indicate significant differences *(p* < 0.05) among groups; Control (C).

Apelin modulates the response of cells to oxidative stress, for example, in rat adipose tissue and adrenal medulla, by reducing the level of markers of oxidative stress [34,36]. In the present study, we measured the direct effect of apelin on oxidative stress in BeWo cells. The cells were incubated with apelin at 2 and 20 ng/mL doses, and after 24 h of oxygen consumption, the assay was performed. We found that both doses of apelin increased the level of fluorescence compared to the control sample, indicating a higher extracellular oxygen consumption upon treatment with apelin (*p* < 0.05, Figure 3).

### 2.4. Involvement of the APJ Receptor and MAP3/1 Kinase in Antiapoptotic Effect of Apelin in BeWo Cells

**Figure 4:**
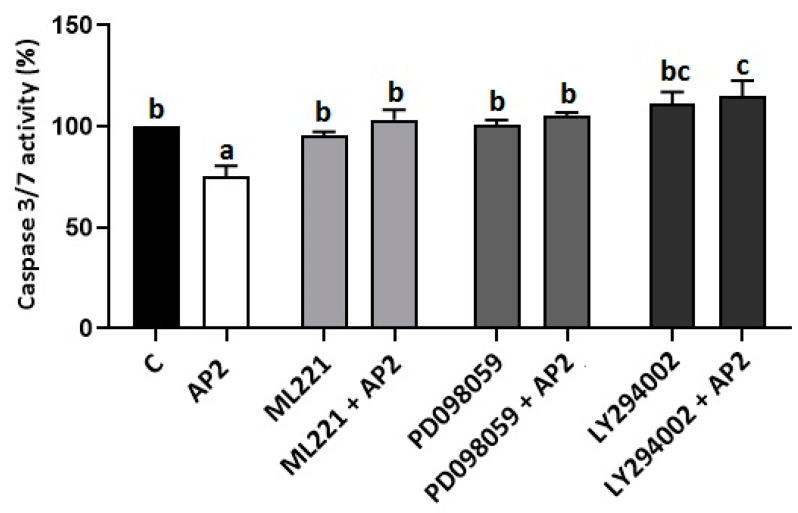
Involvement of APJ receptor, mitogen-activated protein kinase (ERK1/2/MAP3/1) and protein kinase B (AKT) in the antiapoptotic effect of apelin in BeWo cells: The cells were pre-treated for 1 h with APJ receptor, MAP3/1 and AKT kinase inhibitors ML221 (5 µM), PD098059 (1 µM) and LY290042 (1 µM). Subsequently, apelin at a dose of 2 (AP2) ng/mL was added. After 72 h of incubation, activity of caspase 3 and 7 was analysed using the Caspase-Glo 3/7 assay. Experiments were independently performed and repeated three times (*n* = 3). The results are presented as a percentage compared to the control (100%). The data are arranged as means ± SEM. Different letters indicate significant differences (*p* < 0.05) among groups; Control (C).

In our previous study, we have shown that apelin promotes the phosphorylation of MAP3/1 and AKT kinases in BeWo cells [26]. Based on these results and on literature data, we checked the involvement of the APJ receptor and MAP3/1 and AKT kinases in the antiapoptotic effect of apelin BeWo cells. Caspase 3/7 activity was assessed after 72 h of incubation with apelin at 2 ng/mL and pharmacological inhibitors of the APJ receptor and MAP3/1, AKT kinases: ML221 (5 µM), PD098059 (1 µM) and LY290042 (1 µM), respectively. As shown in Figure 4, the activity of caspases 3 and 7 after treatment with 2 ng/mL of apelin was significantly lower compared to that of the control sample, while the simultaneous incubation of placenta cells with ML221, PD098059 and LY290042 added with 2 ng/mL of apelin reversed the antiapoptotic action of apelin (*p* < 0.05, Figure 4).

### 2.5. Effect of Apelin on DNA Fragmentation in Villous Explants from the Third Trimester of Human Pregnancy

**Figure 5:**
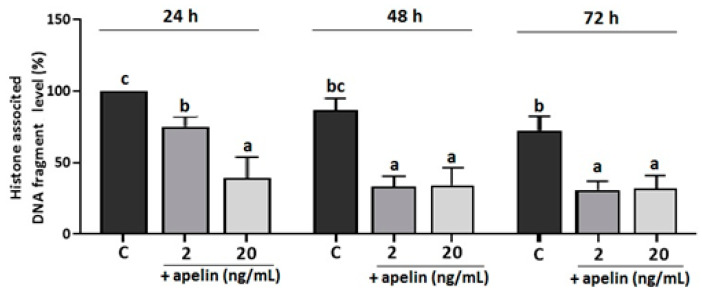
Effect of apelin on DNA fragmentation in villous explants from human placenta. The explants were incubated with apelin at 2 and 20 ng/mL for 24, 48 and 72 h, and the level of histone-associated DNA fragment was measured using the Cell Death Detection ELISA kit. Experiments were performed on five independent cultures of human placenta explants (*n* = 5). The level of DNA fragmentation was calculated to 100 µg protein of villous explants and then as percentage compared to the 24-h control sample (100%). The data are arranged as means ± SEM. Different letters indicate significant differences (*p* < 0.05) among groups; Control (C).

To confirm the results obtained from BeWo cells, we performed additional experiments on primary cultures of villous explants from the third trimester of human pregnancy. Villous explants were incubated with apelin at doses of 2 and 20 ng/mL for 24, 48 and 72 h, and DNA fragmentation was analysed. As shown in Figure 5, apelin significantly decreased the histone-associated DNA fragment level for each incubation time at both apelin doses (*p* < 0.05, Figure 5).

### 2.6. Effect of Apelin on Protein Expression of BCL2, BAX and Caspase-3 in Villous Explants from the Third Trimester of Human Pregnancy

**Figure 6:**
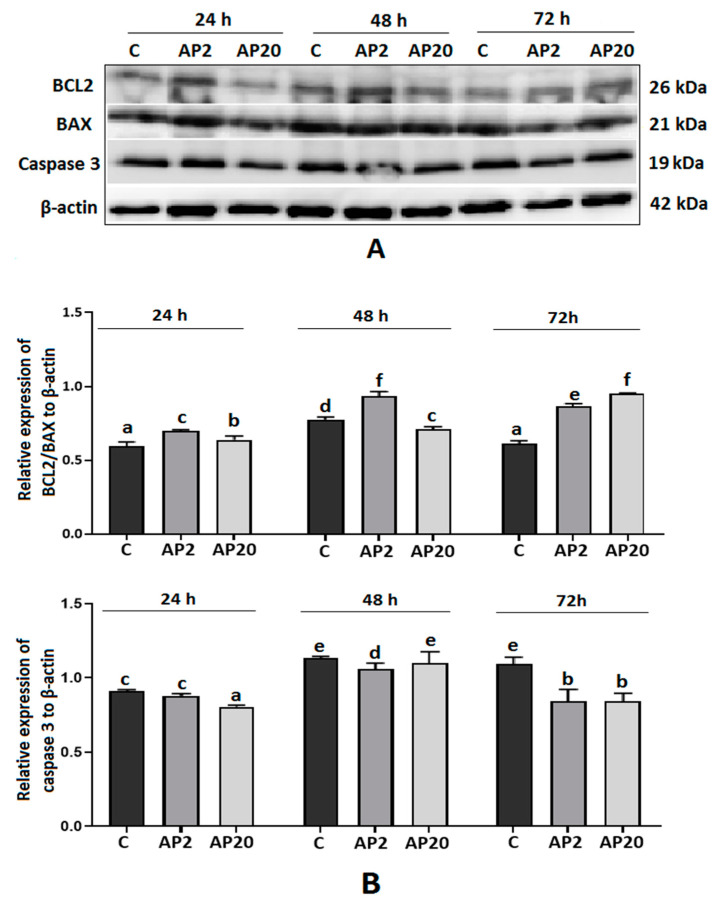
Effect of apelin on protein expression of apoptotic proteins in villous explants from human placenta. The explants were incubated with 2 (AP2) and 20 (AP20) ng/mL of apelin for 24, 48 and 72 h, and Western blot analysis was performed to determine the expression of BCL2, BAX and caspase 3. Results are shown as stripes on gel image (**A**) and densitometry analysis relative to β-actin (**B**). Experiments were performed on five independent cultures of human placenta explants (*n* = 5). The data are arranged as means ± SEM. Different letters indicate significant differences (*p* < 0.05) among groups; Control (C).

As shown in Figure 6, we observed dose- and time-dependent effects of apelin on the protein expression of BCL2, BAX and caspase 3 in villous explants. Apelin significantly upregulated the ratio of BCL2 to BAX after 24 and 48 h of incubation at a dose of 2 ng/mL and after 72 h of incubation at both doses (*p* < 0.05, Figure 6). The protein expression of caspase 3 was significantly lower after treatment with apelin at a dose of 2 ng/mL after 48 and 72 h of incubation, and at 20 ng/mL after 24 and 72 h of incubation (*p* < 0.05, Figure 6).

## 3. Discussion

In the present study, we demonstrate that apelin increased the expression of pro-survival factors and decreased that of proapoptotic factors on mRNA and protein levels in both BeWo cells and villous explants. We also show that apelin significantly inhibits caspase 3/7 activities and DNA fragmentation, also in staurosporine-induced apoptosis, and attenuates oxidative stress by increasing extracellular oxygen consumption. Finally, we prove that the APJ receptor and kinases ERK1/2/MAP3/1 and AKT are involved in the anti-apoptotic effects of apelin in human placenta cells.

The proper functioning of the placenta is one of the critical factors in the maintenance of pregnancy, providing the environment for foetal growth. During placenta development, the following processes must occur: proliferation, invasion, trophoblast differentiation and apoptosis. Non-functional or unnecessary trophoblast cells undergo programmed cell death throughout gestation. To maintain a proper balance between proliferation and apoptosis, many factors must act, such as hormones, proteins, transcription factors and growth factors [1]. As shown in the rat model, the concentration of apelin in maternal circulation is highest in the first trimester and then decreases, which may be due to the higher clearance of this peptide and not the decrease in its secretion [37]. Apelin/APJ expression was described in different parts of the human placenta, among others, in cyto- and syncytiotrophoblasts, the endothelial lining of blood capillaries and the placental artery [26,38,39]. Pyr-apelin 13 is the predominant isoform of apelin in human placental chorionic villi [25]. Thus, we investigated the action of this isoform. So far, the participation of the apelin/APJ system has been studied in the regulation of placenta function, foetal growth, but also in the context of the development and prevention of pregnancy pathologies such as gestational diabetes, preeclampsia or IUGR [37,39,40]. There is substantial evidence of the important role of apelin in pregnancy. Apelin is involved in embryonic angiogenesis, vasodilation [28,29] and the regulation of the endocrine function of the placenta by reducing the secretion of progesterone (P4) and oestradiol (E2) as well as human placental lactogen (hPL) and human chorionic gonadotropin (hCG) [30]. As an important metabolic hormone, it can stimulate trophoblast amino acid and glucose uptake, which indicates its involvement in foetal growth [27,39].

In our previous paper, we showed the expression of the apelinergic system in two human trophoblast cell lines, JEG-3 and BeWo, as well as in human placenta tissue, and described the stimulatory effect of this adipokine on cell cycle progression and the proliferation of trophoblast cells [26]. Nevertheless, there is still a lack of knowledge about the effect of apelin on the apoptosis of these cells. Therefore, in the current research, we focused on the study of potential apelin/APJ involvement in the apoptosis of trophoblast cells. In the first step, we checked the effect of apelin on the expression of numerous factors involved in both main pathways of apoptosis activation. We observed that on the mRNA level, apelin, in a dose-dependent manner, increased the expression of factors promoting cell survival, such as MCL1, BIRC6 XIAP, and BCL3, while it decreased the mRNA of BCL2. In contrast, at the protein level, we noted a stimulatory effect of apelin on the expression of the BCL2 protein. Apelin significantly increased the ratio of pro-survival BCL2 to proapoptotic BAX after 24 h of incubation with 2 ng/mL and after 48 h of incubation with both doses of apelin in BeWo cells. The same effect was observed in villous explants of the human placenta for each incubation time, dependent on the apelin dose. Studies on apoptosis in placentas from uncomplicated term pregnancies and from pregnancies affected by various pathologies have shown that the upregulation of BCL2 expression proves to be crucial for trophoblast survival and for maintaining syncytial integrity. Soni’s group noted a higher apoptotic index in placentas from the first trimester of pregnancy. In contrast, in tissue from the third trimester of gestation, apoptosis was lower, which was associated with higher BCL2 expression in the syncytiotrophoblast [9]. Another study showed that reduced BCL2 expression in the placentas of pregnancies with recurrent miscarriage and those with complicated IUGR and preeclampsia led to the increased death of trophoblast cells and may be one of the etiological components of these diseases [10,41]. Thus, the upregulation of BCL2/BAX expression, observed in our research, is beneficial for placental cells and contributes to their survival.

Our results also show that apelin significantly decreased the expression of proapoptotic factors. At the gene level, after treatment with apelin, we observed lower amounts of transcript for proapoptotic proteins from the BCL2 family, i.e., BAX, BAK1, BOK, proteins involved in death receptor pathway activation (NOD1, CRADD, TNFRSF25), precursors of caspase 2, 3, 8, 9 and 14 and proteins APAF1 and DIABLO. Similarly, the levels of protein of caspase 3, 8 and 9 as well as p53 were lower; they were also time- and dose-dependent. In many placentas from complicated pregnancies, pathologies have been observed with significantly higher expressions of caspase 3 and p53 [10,12,42]. The inhibition of their expression by apelin has a positive effect on trophoblast cell survival. Moreover, in this study, we showed the broad spectrum of apelin’s antiapoptotic effects on human placenta cells and demonstrated that in the placenta, apelin may be involved in the inhibition of many proapoptotic factors involved in both death receptor and mitochondrial pathways.

The activation of effector caspases and the fragmentation of DNA are processes that testify the irreversible entry of the cell into programmed cell death [5]. By examining the effect of apelin on the mentioned processes, we showed that apelin can also inhibit apoptosis by blocking the enzymatic activity of caspase 3 and 7 and the level of histone-associated DNA fragments. Because, in the physiological state, apoptosis frequently occurs in trophoblast cells, particularly in the trophoblast cell line, in this part of the study, we induced apoptosis by staurosporine to compare the action of apelin in a situation when apoptosis occurs at a high rate. Staurosporine is known as an apoptotic agent which leads to cell death by blocking the signalling pathways of several kinases crucial for cell metabolism and survival [43]. It has already been used for the intentional induction of cell death in rat astrocytes, adrenocortical cancer cells or human THP-1 macrophages [43,44,45]. We observed that apelin significantly decreased the enzyme activity of caspase 3 and 7 after 72 h of incubation, also in cells with staurosporine-induced apoptosis. However, we observed no effect on the level of histone-associated DNA fragmentation after the treatment of BeWo cells with apelin alone, but apelin apparently decreased DNA fragmentation in BeWo cells with staurosporine-induced apoptosis at 24 and 48 h of incubation. The results of these experiments confirm the antiapoptotic effect of apelin. Many authors have already highlighted an antiapoptotic effect of apelin in various types of cells, especially in human brain cells, indicating the neuroprotective role of this peptide [46,47]. Moreover, apelin protects rat ovarian cells and adrenal cells from cell death [31,48], which is in agreement with our findings.

One of the harmful factors that can develop in the placenta is hypoxia. The initial anaerobic conditions in the first trimester of pregnancy could support trophoblast development, allowing the proper functioning of integrins that control the proliferation and migration of trophoblast cells [49]. Nevertheless, in the second and third trimesters, the lack or low concentration of oxygen leads to the accumulation of reactive oxygen species (ROS) that damage the tissue. The placenta can respond to chronic hypoxia by increasing the number of apoptotic cells [50]. Progressive oxidative stress and, hence, the apoptosis of the trophoblast cells result in pregnancy pathologies such as IUGR [11]. Our results indicate that apelin may have a beneficial effect on the prevention of apoptosis by the high impact on extracellular oxygen consumption. More specifically, we found that when we used gradually increasing apelin concentrations, BeWo cells effectively maintained their function through reducing oxygen metabolism efficacy. This leads us to infer that apelin attenuates oxidative stress in syncytiotrophoblast placenta cells. Normally, oxygen concentrations at the site of embryo implantation and during the formation of the placenta are low, and this could have a differential effect on the trophoblast cell populations. The effect of apelin on the syncytiotrophoblast cells will be able to attenuate the oxidative stress and to provide the syncytium integrity. However, low oxygen tension is important for the proper function of cytotrophoblasts because it triggers them to proliferate and invade into maternal decidua. Unfortunately, the role of apelin on cytotrophoblast oxygen metabolism efficacy is not described. Nevertheless, the study by Khera et al. [51] showed that after inducing mitochondrial oxidative stress in three cell lines of the human placenta representing different types of placenta cells: choriocarcinoma cells JEG-3—cytotrophoblast and BeWo—syncytiotrophoblast, and also immortalized normal placenta cells Swan-71, cells responded in the same way to selenium treatment, which alleviated the oxidative stress in each of the tested cell types. Although this result was dose-dependent, the biological effect remained the same, and selenium attenuated oxidative stress in trophoblast cell lines, suggesting that regardless of the type and origin of trophoblast cells, their response to the oxidative stress and mitigation factors is similar [51]. In the literature, we also found evidence that apelin acts as an antioxidant; for instance, in the rat adrenal medulla PC12 cell line, it reduced the amount of intracellular ROS, similar as in murine 3T3L1 adipocytes [34,36].

To investigate the molecular mechanism of antiapoptotic apelin action in trophoblast cells, we checked the involvement of the APJ receptor, MAP3/1 and the AKT signalling pathway in apelin-mediated apoptosis. These kinases play a role in signalling pathways involved in proliferation and apoptosis processes [52]. In our previous study, we noted that apelin, in a time-dependent manner, promoted the phosphorylation of these kinases and thus activated them [26]. Based on this knowledge and on available literature data, we hypothesised that apelin can exert antiapoptotic effects in BeWo cells by the activation of MAP3/1 and the AKT signalling pathway. We observed that 2 ng/mL of apelin significantly decreased caspase 3 and 7 activity, while the simultaneous addition of the specific inhibitors of the APJ receptor and the mentioned kinases reversed this effect. More precisely, the antiapoptotic action of apelin was blocked. Based on this, we suggest that the APJ receptor and kinases MAP3/1 and AKT are involved in antiapoptotic effects of apelin in BeWo cells. This is in agreement with previous findings. The activation of MAP 3/1 and AKT signalling pathways and of the APJ receptor is required for the action of antiapoptotic apelin in human vascular smooth muscle cells, mice brains and in the rat adrenal medulla PC12 cell line [31,32,33].

To confirm the results obtained for the human cell line, we performed further experiments on human placenta explants from the third trimester of pregnancy. Placentas were obtained from uncomplicated gestations to investigate the effect of apelin under physiological conditions. In villous explants, apelin exerts potent antiapoptotic effects. It elevates the BCL2/BAX ratio, decreases caspase 3 expression and effectively inhibits DNA fragmentation at each incubation time. Thus, apelin, like leptin, promotes trophoblast survival. Studies on several models of induced apoptosis, high temperature, low pH and serum deprivation conditions in the placenta have shown that in all of them, leptin reduces placental cell death. The authors of these studies demonstrated that through the down-regulation of the p53 pathway and increasing the BCL2/BAX ratio, leptin inhibits apoptosis [16,17,18]. In contrast, adiponectin has the opposite effect and promotes apoptosis in the human placenta by enhancing the expression of BAX, p53 [19,20].

## 4. Materials and Methods

### 4.1. Reagents

Phosphate-buffered saline (PBS), foetal bovine serum (FBS; heat-inactivated), Dulbecco’s Modified Eagle’s Medium (DMEM/F12), trypsin, TaqMan Gene Expression Cell-to-CT Kit (cat. no. AM1728) and the electrophoresis marker were purchased from ThermoFisher Scientific (Waltham, MA, USA). Insulin, glycerol, ethylenediaminetetraacetic acid (EDTA), dithiothreitol, 3,3′-diaminobenzidine (DAB), bromophenol blue, sodium deoxycholate, Nonidet P-40, Tween 20, Laemmli buffer (cat. no. 38733) and human apelin-13 (cat. no. A6469) were obtained from Sigma-Aldrich (St. Louis, MO, USA). The sodium dodecyl sulfate (SDS) and bovine serum albumin (BSA) were purchased from Bioshop Canada, Inc. (Burlington, Canada). Pharmacological blockers ML221 (cat. no. 4748) and PD98059 (cat. no. 1213) were obtained from Tocris Bioscience (Bristol, UK). The WesternBright™ Sirius kit was purchased from Advansta, Inc. (Menolo Park, CA, USA). The Bradford protein assay kit, 4–20% gels (cat. no. 456-1093) and membranes (cat. no. 1704156) were obtained from Bio-Rad Laboratories (Hercules, CA, USA).

### 4.2. In Vitro Culture of BeWo Cells

Choriocarcinoma BeWo cell lines (cat. no. CCL-98) were obtained from the American Type Culture Collection. The BeWo cells were cultured in DMEM/F12 medium without phenol red, supplemented with 0.01 mg/mL insulin and 10% FBS. Cell lines were grown in 75 cm^2^ tissue culture flasks. Cells were seeded in 96-well culture plates in DMEM/F12 with 10% FBS for 24 h at a concentration of 4 × 10^4^ cells per well. Next, the medium was changed to DMEM/F12 with 1% FBS. Cultures were maintained at 37 °C under a humidified atmosphere consisting of 95% O_2_ and 5% CO_2_.

### 4.3. In Vitro Culture of Villous Explants from Human Placenta

Placental tissue was collected in a gynaecological hospital in Krakow, Department of Gynecological Endocrinology, Jagiellonian University Medical College, Poland, where the clinical information on pregnancy outcomes was obtained. Patients gave their informed consent to the study. Clinical information recorded on each pregnancy included the following factors: smoking history, neonatal mortality and pregnancy outcome. Normal term (weeks 40–42 of gestation) placentae from non-smoking and non-endocrinopathies women were collected for the experiment. Immediately after expulsion of the placenta, placental cotyledons were harvested, placed in ice-cold PBS and transported to the laboratory. Placental specimens were obtained (*i*) from the macroscopically normal placenta, (*ii*) from the maternal part, (*iii*) around the central area (not placental periphery), and (*iv*) just 0.5 cm^2^ deep from the maternal surface. The tissue was cut into 10–15 mg pieces, incubated in DMEM/F12 medium in a 24-well plate and maintained at 37 °C in a humidified atmosphere of carbogen gas (95% O_2_ and 5% CO_2_) [16]. Because of the intact structure present in vitro, there is a benefit of culturing placenta pieces. This type of ex vivo culture has been successfully used for ovarian and breast cancer tissue [53,54]. Ovarian tissues have been successfully incubated for up to 50 days without visible signs of necrosis [55].

### 4.4. Experimental Procedure

*Experiment 1*: In the first part of our study, we focused on examining the apelin effect on oxidative stress and apoptosis in human BeWo cell line. First, BeWo cells were cultured in DMEM F12 medium supplemented with 1% FBS and containing apelin at concentrations of 2 and 20 ng/mL. Doses of apelin were chosen based on our previous paper [26]. After 24, 48 and 72 h, cells were washed in PBS and stored at −20 °C for analysis of protein expression of BCL2, BAX, caspase 3, 8, 9 and p53 by Western blot. After 24 h of incubation, cells were washed in PBS and stored at −70 °C for further analysis of mRNA expression of multiple apoptotic genes by real-time PCR or to determine extracellular oxygen consumption using the MitoXpress-Xtra Oxygen Consumption assay.

*Experiment 2:* In the following experiments, we investigated the apelin effect on caspase 3 and 7 enzyme activity and DNA fragmentation in BeWo cells in physiological conditions and after induction of apoptosis. The BeWo cells were cultured in DMEM F12 medium supplemented with 1% of FBS and apelin at doses of 2 and 20 ng/mL alone or in combination with 0.1 ng/mL of staurosporine to induce apoptosis for 24, 48 and 72 h. Then, the Caspase-Glo 3/7 assay was performed to investigate caspase 3 and 7 activity or cells were washed in PBS and stored at −70 °C for analysis of DNA fragmentation using the Cell Death Detection ELISA kit.

*Experiment 3:* To investigate the molecular mechanism of the antiapoptotic effect of apelin in BeWo cell line, cells were preincubated for 1 h with DMEM F12 medium supplemented with 1% FBS and pharmacological blockers of APJ receptor and kinases MAP3/1 and AKT; ML221 (5 µM), PD098059 (1 µM) and LY290042 (1 µM), respectively. Doses of blockers were selected based on our previous data and preliminary experiments [26]. Next, 2 ng/mL of apelin was added, and after 72 h, the Caspase-Glo 3/7 assay was performed to determine caspase 3 and 7 activity.

*Experiment 4:* To confirm the results obtained in the BeWo cell line, we performed additional experiments on explants of human placenta. Villous explants were cultured in DMEM F12 medium supplemented with 1% of FBS and containing 2 or 20 ng/mL of apelin for 24, 48 and 72 h. After incubation, explants were washed in PBS, lysed, and cell lysates were stored at −70 °C to determine DNA fragmentation using the Cell Death Detection ELISA kit or at −20 °C to analyse protein expression of BCL2, BAX and caspase 3.

### 4.5. Real-Time PCR

Total RNA isolation and cDNA synthesis were performed using the TaqMan Gene Expression Cell-to-CT Kit (cat. no. 4399002, Applied Biosystems, Carlsbad, CA, USA) according to the manufacturer’s protocol. Amplifications were performed using StepOne Real-Time PCR (Applied Biosystems, Carlsbad, CA, USA). TaqMan Array Human Apoptosis (nr kat. 4414072, Applied Biosystems, Carlsbad, CA, USA) containing specific primers for genes of apoptotic factors (Table 2) and GAPDH, 18S as internal control was used.

Quantitative PCR was performed with 100 ng cDNA, 1 mL TaqMan Gene Expression primers and 10 mL TaqMan PCR master mix (Applied Biosystems, Carlsbad, CA, USA) in a final reaction volume of 20 mL. The thermal cycling conditions were as follows: 50 °C for 2 min, 95 °C for 10 min and 40 cycles of 95 °C for 15 s and 60 °C for 1 min to determine the cycle threshold number (Ct) for quantitative measurement. The relative mRNA expression levels of apoptosis genes relative to GAPDH and 18S were determined using the 2^−ΔΔCq^ method [56].

### 4.6. Western Blot Analysis

The Western blot procedure used to determine BCL2/BAX, caspase 3, 8, 9 and p53 proteins expression has been described previously [26]. Briefly, equal amounts of BeWo cells and villous explants lysates (50 μg protein/sample) were separated by 4–20% Mini-Protean TGX System Precast Protein Gels and transferred to Trans-Blot Turbo Mini PVDF Transfer Packs (Bio-Rad Laboratories, Hercules, CA, USA). The membranes were blocked for 1 h in 0.02 M Tris-buffered saline containing 5% BSA and 0.1% Tween 20 and then incubated overnight at 4 °C with primary antibody for BCL2 (cat. no. #2876, Cell Signaling Technology, Danver, MA, USA (CST), 1:1000), BAX (cat. no. #2772, CST, 1:1000), caspase 3 (cat. no. #9662, CST, 1:1000), caspase 8 (cat. no. MA1-41280, ThermoFisher, Waltham, MA, USA, 1:500), caspase 9 (cat. no. PA5-22252, ThermoFisher, 1:500) and p53 (cat. no. #9282, CST, 1:1000). Subsequently, the membranes were washed with TBST (Tris-buffered saline containing 0.1% Tween 20) and incubated for 1 h at room temperature with horseradish peroxidase-conjugated anti-rabbit (cat. no. #7074, CST) or anti-mouse (cat. no. #7076, CST) antibody diluted at 1:1000. Signals were detected by chemiluminescence using the Western blotting Luminol Reagent (cat. no. K-12043 D20, Advansta Inc., Menlo Park, CA, USA) and visualised using the Chemidoc XRS+ System (BioRad Laboratories, Hercules, CA, USA). The blots were stripped and probed for anti β-actin as a loading control. All visible bands were quantified using a densitometer and the ImageJ 1.44 software (US National Institutes of Health, Bethesda, MD, USA).

### 4.7. Extracellular Consumption Assay

The extracellular oxygen consumption by BeWo cells was measured on a multi-mode microplate filter reader (FLUOstar OPTIMA, BMG Labtech, Offenburg, Germany), using the MitoXpress-Xtra Oxygen Consumption assay (cat. no. HS-100D-1 Agilent Technologies, Inc. Santa Clara, CA, USA) in accordance with the manufacturer’s protocol. Fluorescence intensity was measured in 10 min intervals for 90 min at 37 °C under a sealed environment by overlaying with 100 μL of mineral oil to limit oxygen exchange. The rate of change in fluorescence signal per minute was calculated for each 10 min interval, and an average signal change per minute during the whole 90 min period was calculated for each cell line. Traces of fluorescence were transformed into oxygen concentration profiles based on the work of Konieczka et al. [57], using the following Equation (1):

[O_2_] (t) = [O_2_] × Ia × (Io − I (t)) I (t) × (Io − Ia) (1)

where [O_2_] is the oxygen concentration in the air-saturated buffer (235 μM at 30 °C); I (t), Ia and Io are the probe fluorescent signal at time t, the signal in air-saturated buffer (baseline signal without enzyme) and the signal in deoxidised buffer (maximum signal), respectively. The dissolved oxygen change rate was then determined from the initial slopes of these concentration profiles.

### 4.8. Caspase-Glo 3/7 Assay

The Caspase-Glo 3/7 assay (cat. no. G8090, Promega, Madison, WI, USA) was used to check caspase 3 and 7 activity. The addition of Caspase-Glo 3/7 reagent resulted in cell lysis, followed by caspase cleavage of the substrate. The substrate released aminoluciferin, which was consumed by luciferase and then generated a “glow-type” luminescent signal that is proportional to caspase 3 and 7 activity. The Caspase-Glo 3/7 assay solution was aseptically added to wells in amounts equal to 100% [*v/v*] of the incubation volume. After 1.5 h of incubation, absorbance was measured at 495 nm wavelength using a luminometer SpectraMax L 147 with the SoftMax Pro software (v. 7, Molecular Devices, San Jose, CA, USA).

### 4.9. Cell Death Detection ELISA Kit

The Cell Death Detection ELISA kit (cat. no. 11 544 675 001, Roche Diagnostics, Basel, Switzerland) is an enzyme immunoassay used for the determination of levels of cytoplasmic histone-associated DNA fragment (mono and oligonucleosomes). The assay is based on the quantitative sandwich enzyme immunoassay principle using mouse monoclonal antibodies directed against DNA and histones. The assay was performed according to the manufacturer’s protocol. Absorbance was measured at the 405-nm wavelength using an ELx808 ELISA microplate reader and the KC JUNIOR software (BioTek Instruments, Winooski, VT, USA).

### 4.10. Statistical Analysis

All experimental data were presented as the means ± standard error of the mean (SEM) of a minimum of three independent experiments for BeWo cells (*n* = 3). In the case of villous explants, each experiment was performed on five independent cultures of human placenta explants (*n* = 5). Normality was checked by the Shapiro–Wilk test. One-way ANOVA was used to determine differences among more than two treatment groups, and the Tukey test was used post-hoc (GraphPad Prism 8 Software; La Jolla, CA, USA). Statistical significance is indicated by different letters (*p* < 0.05): the same letters indicate no significant difference, with a < b < c < d < e < f or * *p* < 0.05, ** *p* < 0.1, *** *p* < 0.01.

## 5. Conclusions

**Figure 7:**
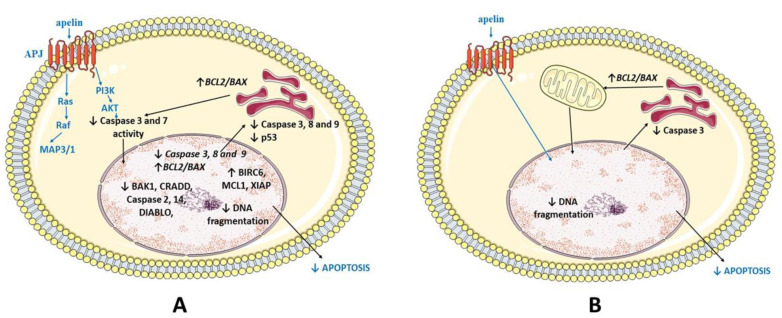
Model of apelin antiapoptotic action in BeWo cell line (**A**) and human placenta explants (**B**). Apelin inhibits apoptosis by activation of apelin receptor (APJ), mitogen-activated kinase (MAP3/1) and protein kinase B (AKT); BCL2 (B-cell lymphoma 2), BAX (Bcl-2-like protein 4); BAK1 (Bcl-2 homologous antagonist/killer); BOK (Bcl-2-related ovarian killer protein); NOD1 (Nucleotide-binding oligomerisation domain-containing protein 1); CRADD (Caspase and RIP adapter with death domain); DIABLO (Diablo IAP-Binding Mitochondrial Protein); TNFRSF25 (Tumour necrosis factor receptor superfamily member 25); BIRC6 (Baculoviral IAP repeat-containing protein 6); MCL1 (Induced myeloid leukaemia cell differentiation protein MCL1); XIAP (X-linked inhibitor of apoptosis).

Based on our results, we suggest that apelin, by increasing the mRNA and protein expression of pro-survival factors and decreasing proapoptotic agents, inhibiting caspase 3 and 7 enzyme activity as well as DNA fragmentation, has antiapoptotic effects in the human placenta (Figure 7). Taking into account our previous research, indicating the stimulatory effect of apelin on trophoblast cell proliferation, we suggest that apelin promotes trophoblast survival and is an important regulator of human placenta development.

## Figures and Tables

**Table 1:** Effect of apelin on mRNA expression of apoptotic factors in BeWo cells. The cells were incubated with apelin at doses 2 (AP2) and 20 ng/mL (AP20) for 24 h, after which real-time PCR analysis was performed. Experiments were independently performed and repeated minimum three times (*n* = 3).

Assay ID	Description of Gene	Gene Symbol	Average Fold Change	
			AP2	AP20
**Pro-Survival Protein**				
Hs00608023_m1	B-cell lymphoma 2	*BCL2*	0.26 **	1.09
Hs00180403_m1	B-cell lymphoma 3 protein	*BCL3*	0.44 ***	2.00 **
Hs00212288_m1	Baculoviral IAP repeat-containing protein 6	*BIRC6*	1.77 **	0.99
Hs00172036_m1	Induced myeloid leukemia cell differentiation protein Mcl-1	*MCL1*	1.48 *	1.12
Hs00745222_s1	X-linked inhibitor of apoptosis	*XIAP*	0.94	2.08 ***
**Pro-Apoptotic Protein**				
Hs00559441_m1	Apoptotic protease-activating factor 1 (Apaf-1)	*APAF1*	0.93	0.77 *
Hs00832876_g1	Bcl-2 homologous antagonist/killer	*BAK1*	0.26 **	0.47 **
Hs00751844_s1	Bcl-2-like protein 4	*BAX*	0.80 *	0.45 **
Hs00609632_m1	BH3-interacting domain death agonist	*BID*	1.02	0.98
Hs00261296_m1	Bcl-2-related ovarian killer protein	*BOK*	0.38 **	1.17
Hs00196075_m1	Nucleotide-binding oligomerization domain containing protein 1	*NOD1*	0.61 *	0.60 *
Hs01011159_g1	Caspase and RIP adapter with death domain	*CRADD*	0.74 *	0.94
Hs00201637_m1	Caspase 14 precursor	*CASP14*	0.58 ***	0.68 **
Hs00892481_m1	Caspase 2 precursor	*CASP2*	0.80 ***	0.51 ***
Hs00234387_m1	Caspase 3 precursor	*CASP3*	0.59 **	0.58 **
Hs01018151_m1	Caspase 8 precursor	*CASP8*	0.59 *	0.63 *
Hs00154260_m1	Caspase 9 precursor	*CASP9*	1.09	0.57 *
Hs00172768_m1	Death effector domain containing	*DEDD*	1.12	0.97
Hs00219876_m1	Diablo IAP-binding mitochondrial protein	*DIABLO*	0.89	0.87 *
Hs00980365_g1	Tumor necrosis factor receptor superfamily member 25	*TNFRSF25*	0.51 **	0.57 *

The data are arranged as means ± standard error of the mean (SEM). Significance between control and apelin treatments is indicated by * *p* < 0.05, ** *p* < 0.01, *** *p* < 0.001. Control value is 1.

**Table 2:** Characteristics of investigated apoptosis genes.

Gene Symbol	Gene Name	Catalog Number	Reference Sequence
*BCL2*	B-cell lymphoma 2	Hs00608023_m1	NM_000633.2
*BCL3*	B-cell lymphoma 3 protein	Hs00180403_m1	NM_005178.4
*BIRC6*	Baculoviral IAP repeat-containing protein 6	Hs00212288_m1	NM_016252.3
*MCL1*	Induced myeloid leukemia cell differentiation protein Mcl-1	Hs00172036_m1	NM_001197320.1
*XIAP*	X-linked inhibitor of apoptosis	Hs00745222_s1	NM_001167.3
*APAF1*	Apoptotic peptidase-activating factor 1	Hs00559441_m1	NM_013229.2
*BAK1*	Bcl-2 homologous antagonist/killer	Hs00832876_g1	NM_001188.3
*BAX*	Bcl-2-like protein 4	Hs00751844_s1	NM_001291428.1
*BID*	BH3-interacting domain death agonist	Hs00609632_m1	NM_197966.2
*BOK*	Bcl-2-related ovarian killer protein	Hs00261296_m1	NM_032515.4
*NOD1*	Nucleotide-binding oligomerization domain containing protein 1	Hs00196075_m1	NM_006092.2
*CRADD*	Caspase and RIP adapter with death domain	Hs01011159_g1	NM_001320099.1
*CASP14*	Caspase-14 precursor	Hs00201637_m1	NM_012114.2
*CASP2*	Caspase-2 precursor	Hs00892481_m1	NM_001224.4
*CASP3*	Caspase-3 precursor	Hs00234387_m1	NM_004346.3
*CASP8*	Caspase-8 precursor	Hs01018151_m1	NM_001228.4
*CASP9*	Caspase-9 precursor	Hs00154260_m1	NM_001229.4
*DEDD*	Death effector domain containing	Hs00172768_m1	NM_001039711.1
*DIABLO*	Diablo IAP-Binding Mitochondrial Protein	Hs00219876_m1	NM_001278302.1
*TNFRSF25*	Tumor necrosis factor receptor superfamily member 25	Hs00980365_g1	NM_003790.2

## Data Availability

The data presented in this study are available on request from the corresponding author.

## Abbreviations

AKT	Protein kinase B
AP2	Apelin at dose 2 ng/mL
AP20	Apelin at dose 20 ng/mL
APAF1	Apoptotic protease-activating factor 1
BAK	Bcl-2 homologous antagonist/killer
BAX	Bcl-2-like protein 4
BCL2	B-cell lymphoma 2
BCL3	B-cell lymphoma 3 protein
BID	BH3-interacting domain death agonist
BIRC6	Baculoviral IAP repeat-containing 6
BOK	Bcl-2-related ovarian killer protein
BSA	Bovine Serum Albumin
CASP 7	Caspase 7
CASP14	Caspase 14
CASP2	Caspase 2
CASP3	Caspase 3
CASP8	Caspase 8
CRADD	Caspase and RIP adapter with death domain
CST	Cell Signaling Technology
Ct	Cycle threshold number
DAB	3,3′-diaminobenzidine
DEDD	Death effector domain containing
DIABLO	Diablo IAP-binding mitochondrial protein
DMEM	Dulbecco’s Modified Eagle’s Medium
E2	Oestradiol
EDTA	ethylenediaminetetraacetic acid
ERK1/2/MAP3/1	Mitogen-activated protein kinase
FBS	Fetal Bovine Serum
hCG	Chorionic gonadotropin
hPL	Human placental lactogen
IUGR	Intrauterine growth restriction
MCL-1	Induced myeloid leukemia cell differentiation protein MCL1
NOD1	Nucleotide-binding oligomerization domain containing protein 1
P4	Progesterone
PBS	Phosphate-buffered saline
pyr-apelin-13	Pyroglutamylated apelin-13
RAU	Relative Absorbance Unit
RLU	Relative Luminescence Unit
ROS	Reactive oxygen species
SDS	sodium dodecyl sulfate
SEM	Standard error of the mean
TNFR	Tumor necrosis factor receptor
TNFRSF25	Tumor necrosis factor receptor superfamily member 25
XIAP	X-linked inhibitor of apoptosis
